# Surgical treatment of a very rare case of a huge intraligamental leiomyoma of the uterus: A case report and mini‑review of the literature

**DOI:** 10.3892/mi.2023.126

**Published:** 2023-12-01

**Authors:** Anna Thanasa, Efthymia Thanasa, Evangelos Kamaretsos, Vasiliki Grapsidi, Emmanouil Xydias, Apostolos Ziogas, Evangelos-Ektoras Gerokostas, Ioannis-Rafail Antoniou, Ioannis Paraoulakis, Ioannis Thanasas

**Affiliations:** 1 Department of Health Sciences, Medical School, Aristotle University of Thessaloniki, 54124 Thessaloniki, Greece; 2 Department of Obstetrics and Gynecology, General Hospital of Trikala, 42100 Trikala, Greece; 3 Department of Obstetrics and Gynecology, EmbryoClinic IVF, 55133 Thessaloniki, Greece; 4 Department of Medicine, University of Thessaly, School of Health Sciences, 41334 Larissa, Greece

**Keywords:** intraligamental leiomyoma, computed tomography, magnetic resonance imaging, surgical treatment, electrothermal bipolar vessel sealing device, intraoperative complications

## Abstract

Intraligamental leiomyomas of the uterus are rare. Extremely rare are the huge intraligamental fibroids (>20 cm), whose pre-operative diagnosis and surgical management poses a challenge to everyday clinical practice. The present study describes the case of patient who was subjected to surgical treatment for a huge intraligamental leiomyoma of the uterus, which weighed 3,370 g. A 48-year-old patient, without menstrual disorders and with a medical history of atypical symptoms from the digestive tract, was referred for a gynecological examination. Upon a physical examination, the abdomen was found to be bloated and distended, with no signs of peritoneal irritation. An intra-abdominal mass was suspected, the upper margin of which was palpable at about the level of the xiphoid process. The findings of computed tomography and magnetic resonance imaging confirmed the presence of a huge intra-abdominal mass, which probably originated from the internal genital organs. Following consultation with the patient, surgical treatment with laparotomy was decided. Intraoperatively, a large pedunculated subserosal leiomyoma was found, arising from the right lateral wall of the uterus with retroperitoneal extension within the leaves of broad ligament. Following the resection of the intraligamental leiomyoma, which had significant surgical challenges, a total hysterectomy with bilateral adnexectomy was performed. The post-operative course was smooth. In addition, in the present study, a brief review of intraligamental leiomyomas of the uterus is presented, emphasizing the significant diagnostic and surgical challenges and potential intraoperative complications that may arise in the management of patients with this condition.

## Introduction

The term fibroid was first introduced into everyday clinical practice in the 1860s. Fibroids or leiomyomas are benign neoplasms that grow on the uterine wall in women of reproductive age and are currently the most common gynecological tumors worldwide (1). Depending on their development in the myometrium and their location relative to the uterine cavity, leiomyomas can be divided into submucosal, intramural and subserosal (2). Among the above, three main types are leiomyomas located outside the uterus corpus, such as the cervix (cervical leiomyomas) and the broad ligament (intraligamental leiomyomas) (3).

Intraligamental leiomyomas are pedunculated tumors, which originate from the lateral wall of the uterus corpus and grow within the broad ligaments. Intraligamental leiomyomas are rare. They are estimated to account for 6 to 10% of all uterine fibroids (4). Huge intraligamental leiomyomas (>10 cm) are even rarer. Extremely rare are the huge intraligamental leiomyomas (>20 cm). It is estimated that to date, five cases of huge intraligamental leiomyomas have been reported in the English literature, of which the maximum diameter was >20 cm and the weight was >3 kg (5-8).

The present study describes the case of patient who was subjected to surgical treatment for a huge intraligamental leiomyoma of the uterus. It is pointed out that, despite the rarity of the nosological entity concerning tumors >3 kg and the serious difficulties in differential diagnosis it presents from other intra-abdominal tumors, the adequate pre-operative preparation of the patient is considered necessary in order to ensure the optimal intraoperative and post-operative outcomes. At the same time, the significant surgical challenges and potential intraoperative complications that may arise in the treatment of huge intraligamental leiomyomas of uterus are highlighted.

## Case report

The present study describes the case of a 48-year-old patient, who visited the Gynecological Outpatient Clinic of the General Hospital of Trikala (Trikala, Greece) for an examination. The patient presented an abdominal ultrasound, which was performed upon the recommendation of the general practitioner. To the family physician, the patient complained of abdominal distension with atypical abdominal pain, dyspepsia and anorexia. Ultrasound imaging revealed the presence of a space-occupying lesion of mixed echogenicity and an enormous size, which occupied most of the peritoneal cavity and was most likely originating from the right ovary. Furthermore, after obtaining a comprehensive medical history, it was revealed that the aforementioned symptoms first appeared ~20 months ago, during which time the patient had not requested medical care until she visited her family physician. In addition, from her personal medical history, hypothyroidism was reported, although it was well regulated with medication. Her menstrual cycle was normal. The patient had given birth to a child by vaginal delivery 15 years prior.

**Figure 1:**
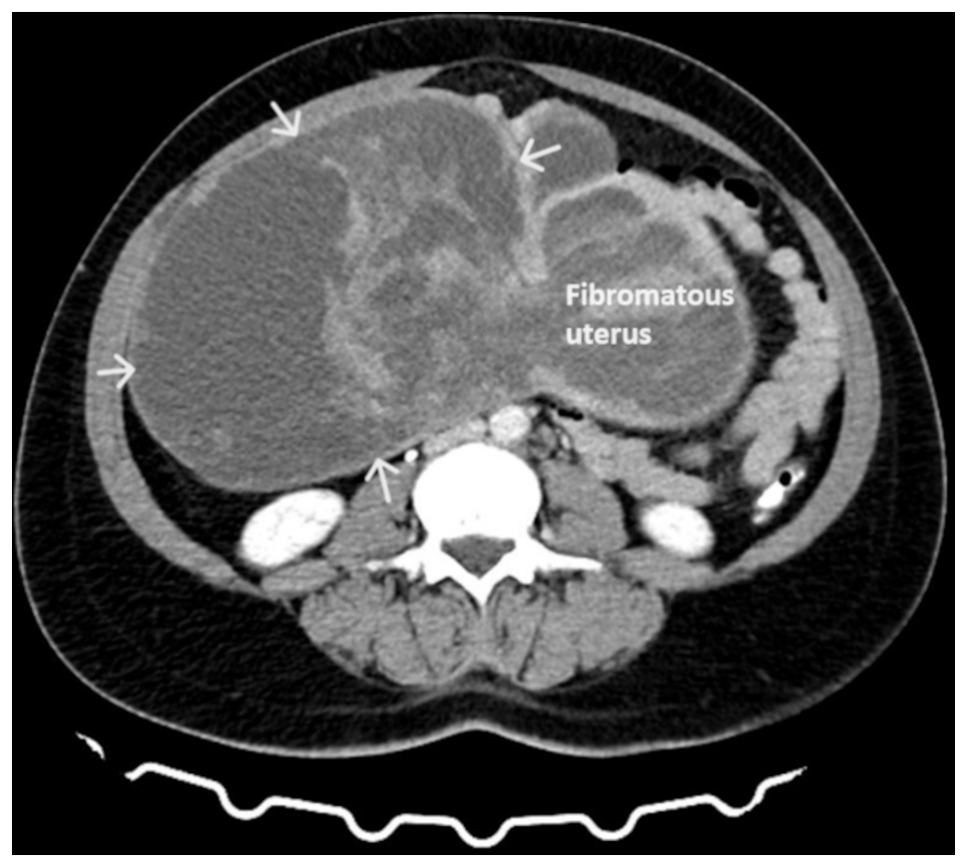
Computed tomography scan illustrating a huge intrauterine leiomyoma of the uterus: A large, mixed echogenicity mass with well-defined margin (white arrows) is evident, occupying most of the peritoneal cavity

**Figure 2:**
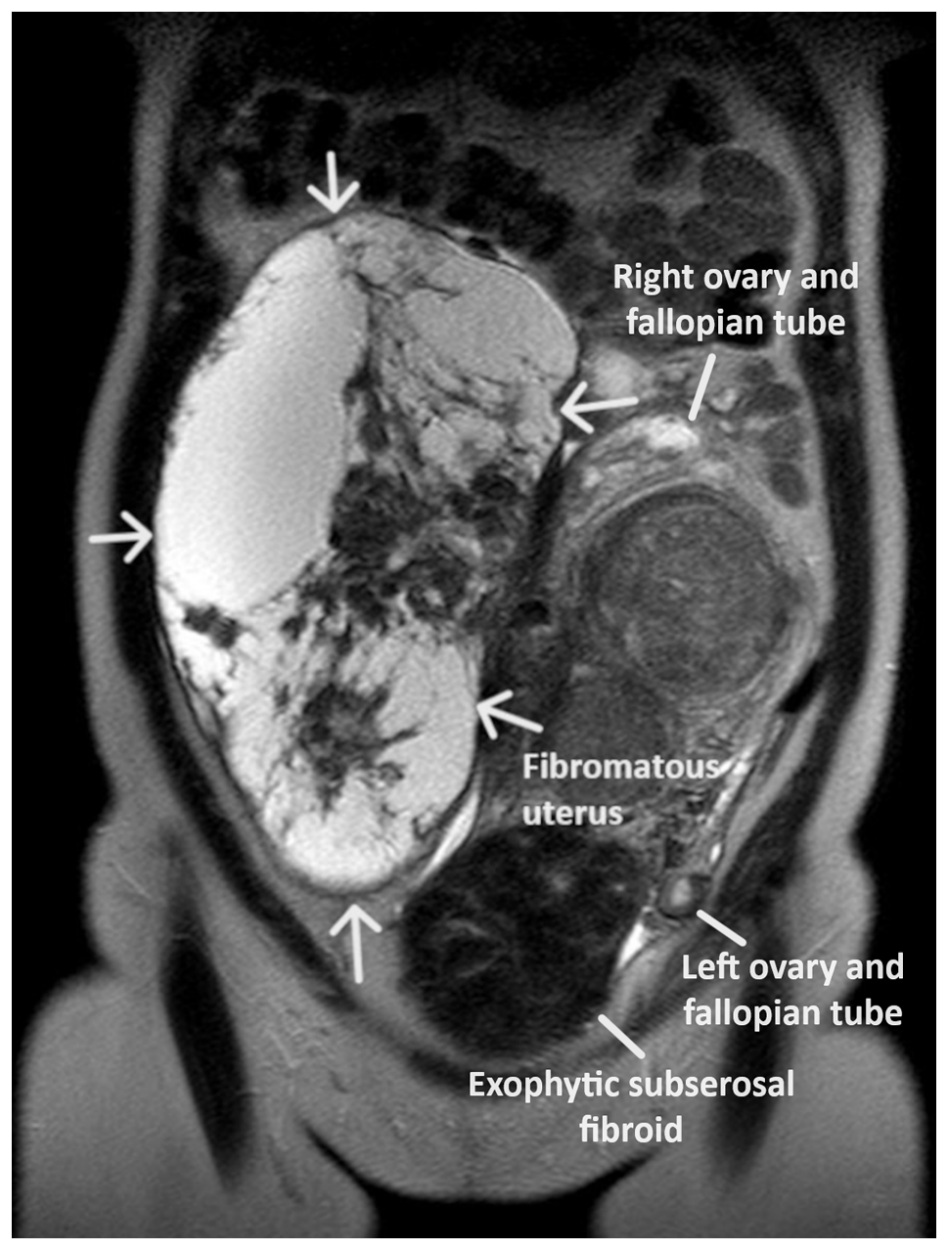
Magnetic resonance imaging of a huge intraligamental leiomyoma of the uterus: The space-occupying lesion is depicted by white arrows with lobulated margins and extensive nodular solid and cystic necrotic elements, nodular wall protrusions and multiple internal septations; it corresponds to a uterine leiomyoma with growth within the broad ligament.

Upon a physical examination, the abdomen was found to be bloated and rigid, with no signs of peritoneal irritation. The upper margins of the tumor were palpable at approximately the level of the xiphoid process. A Visual inspection of the cervix was difficult due to its displacement by the intra-abdominal mass. The transvaginal ultrasound was not diagnostic. A computed tomography scan revealed the presence of a large mass (28.5x23x11 cm), with well-defined margins, which occupied most of the peritoneal cavity and probably of adnexal origin. The intra-abdominal mass had a multilobulated shape and was of mixed consistency, with marked post-contrast enhancement of solid elements (Fig. 1). The findings from magnetic resonance imaging were similar. Magnetic resonance imaging revealed a large heterogeneous lesion measuring ~26x13 cm, which originated from the internal genital organs. As a consequence of the large size, the lesion exited from the lesser pelvis and extended superiorly, completely occupying the peritoneal cavity of the mid-abdomen up to approximately the middle of the right kidney, with a maximum cephalocaudal length of 27.5 cm. The lesion was depicted with lobulated indistinct borders and had extensive nodular solid and cystic necrotic elements, nodular wall protrusions and multiple internal septations, presenting inhomogeneous enhancement in the paramagnetic substance (Fig. 2). The present imaging depicted a part of the uterus, which appeared to be encapsulated and indistinct within the lesion, with the presence of multiple solid inhomogeneous formations mimicking fibroids. The left ovary was displaced in the left iliac fossa with the presence of small follicles. The right ovary was not visualized. From the laboratory analysis, the following results were obtained: Hematocrit, 29.8%; hemoglobin, 9.1 g/dl; white blood cell count, 9.820/ml; neutrophils, 63.2%; platelets, 271x103/ml; international normalized ratio, 0.99; fibrinogen, 278 mg/dl; urea, 34 mg/dl; creatinine, 0.71 mg/dl. The cervical smear test was negative for malignancy. The levels of tumor markers [carcinoembryonic antigen, cancer antigen (Ca)125, Ca15-3 and Ca19-9] were within normal range.

**Figure 3:**
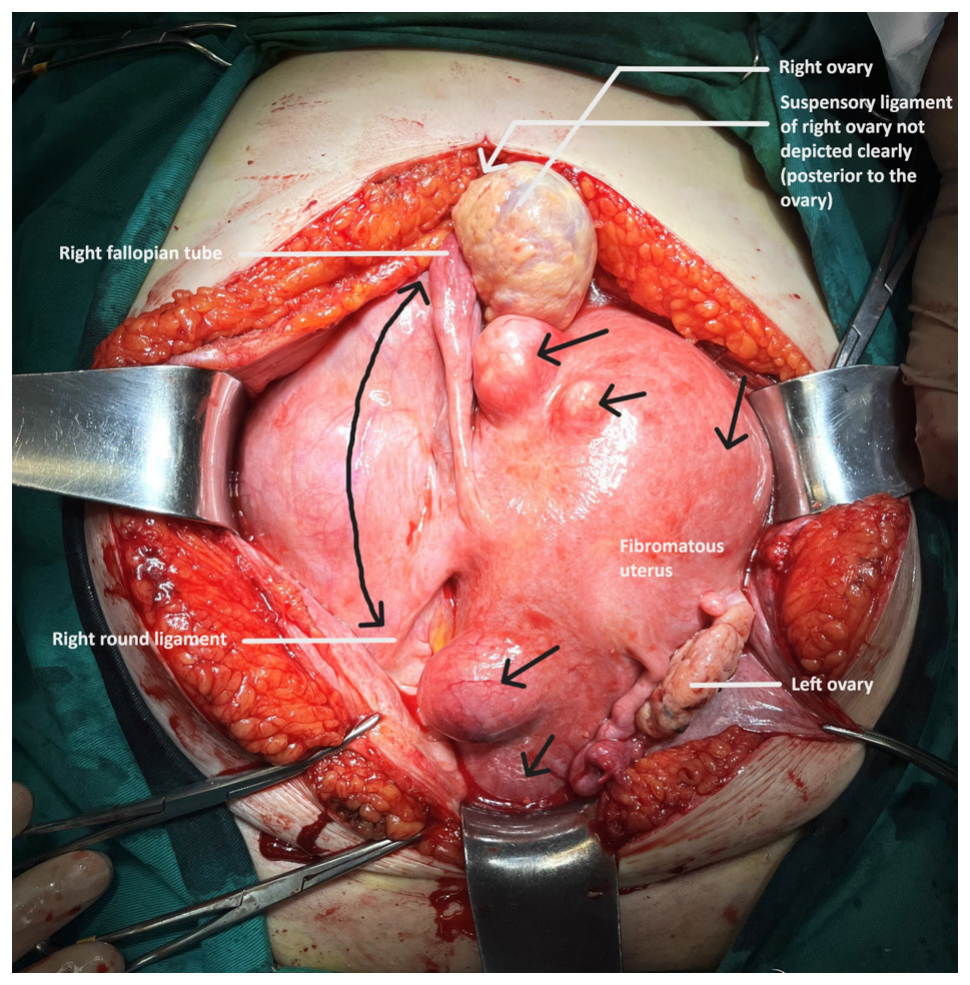
Intraoperative image of a huge intraligamental leiomyoma of the uterus: The extension of the tumur into the retroperitoneal space within the leaves of the broad ligament is evident. At the same time, the presence of multiple subserosal and intramural uterine leiomyomas (black arrows) and the displacement of both ovaries are depicted.

**Figure 4:**
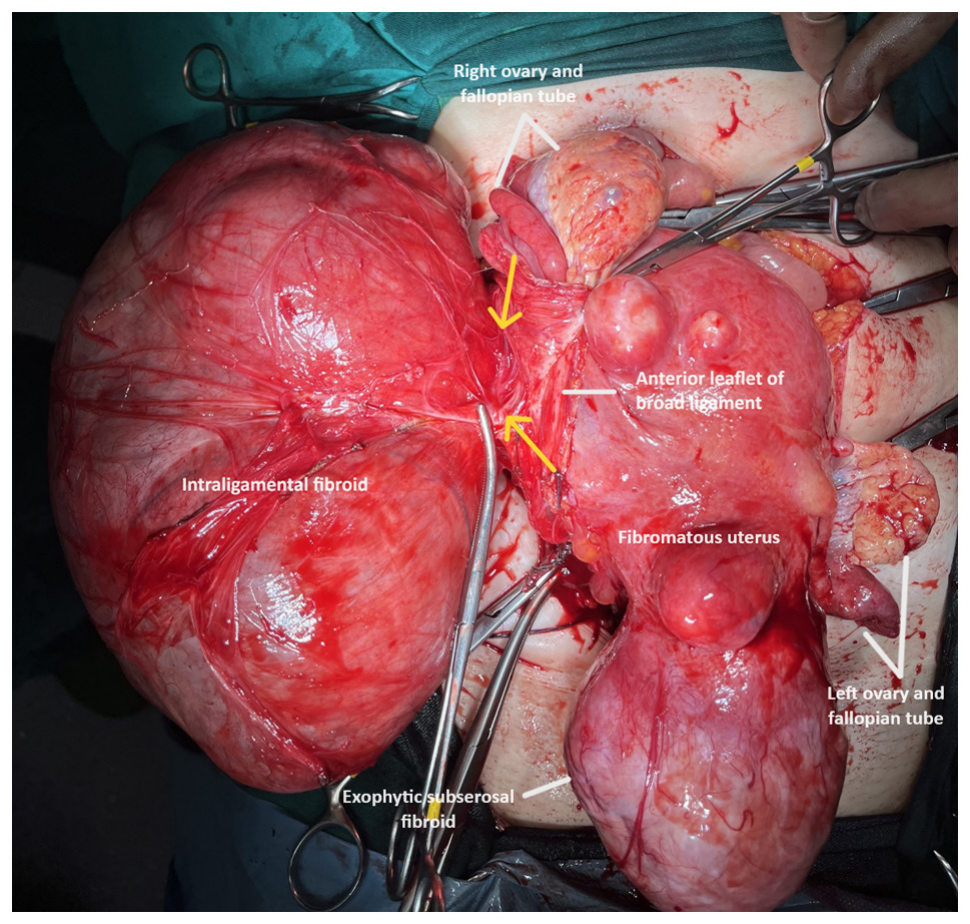
Intraoperative image of a huge intraligamental leiomyoma of the uterus, originating (yellow arrows) from the right lateral wall of the uterus.

**Figure 5:**
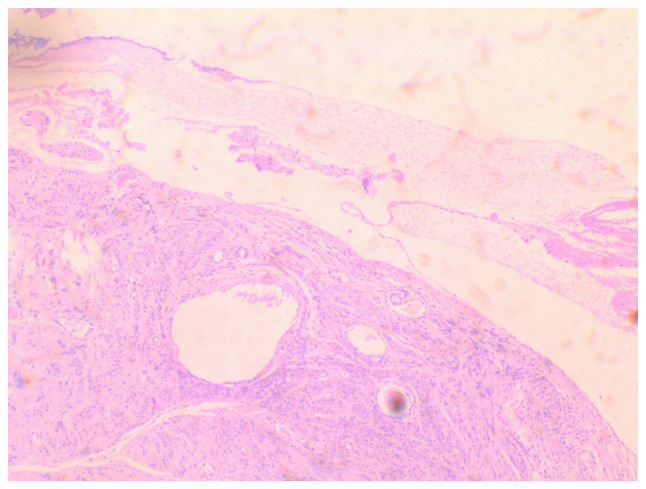
Histological image of intraligamental leiomyoma of the uterus: Leiomyoma with cystic and hydropic degeneration is shown (hematoxylin and eosin staining; magnification, x40).

**Figure 6:**
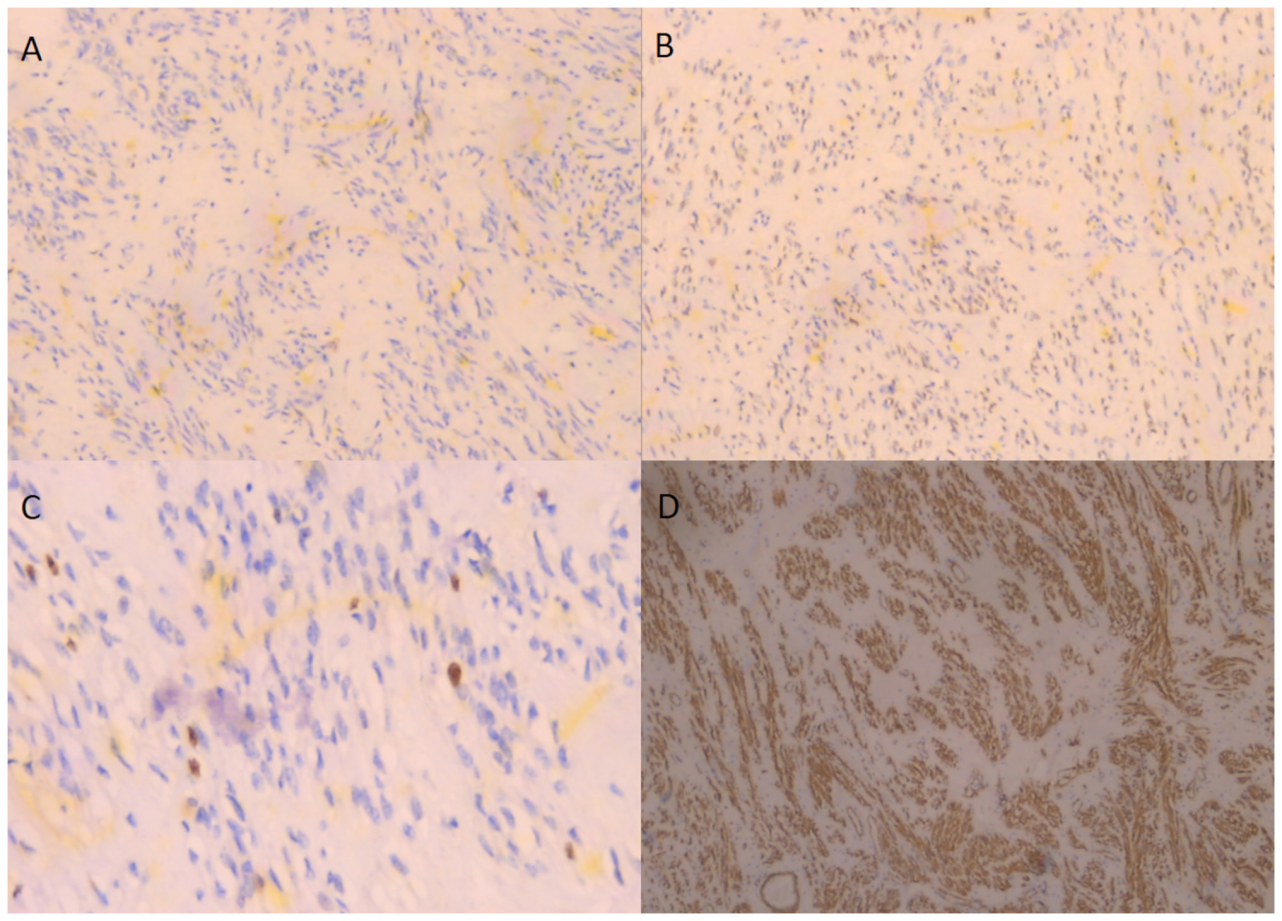
Immunohistochemical images of leiomyoma illustrating: (A) CD10 negativity (magnification, x100), (B) estrogen receptor (ER) focal weak positivity (magnification, x100), (C) Ki67, 7% (magnification, x400), (D) smooth muscle actin positivity (magnification, x40).

Following a thorough consultation of the patient, it was decided to perform an exploratory laparotomy, with the possible necessity of performing a total abdominal hysterectomy. Pre-operatively, ureteral stents were placed. Intraoperatively, a large subserosal pedunculated leiomyoma was found emerging from the right lateral wall of the uterus with retroperitoneal extension within the leaves of the broad ligament. The ovaries were displaced. The presence of other smaller uterine fibroids was evident (Fig. 3). Using an electrothermal bipolar vessel sealing device (LigaSure™), a dissection of the anterior leaf of the broad ligament was performed, from the upper third of the round ligament to the suspensory ligament (both ligaments had been previously ligated with a suture). Subsequently, with the use of the electrothermal bipolar vessel sealing device and using suture ligation where necessary, the fibroid (weighing 3,370 g) was dissected from the leaves of the broad ligament and resected following the ligation and dissection of the vascular pedicle in the lateral right wall of uterus (Fig. 4). Surgical steps were carefully performed to avoid injury to the ureters, bladder and the large vessels, and their branches that pass through the anatomical area. The surgery was completed with the resection of the uterus and adnexa. Transfusion with two units of whole blood was deemed necessary. A histological examination of the surgical specimen confirmed the diagnosis of intraligamental leiomyoma of the uterus. A microscopic examination of the tumor revealed cystic and hydropic degeneration with a low mitotic index (0 to 2 mitoses) and thick-wall blood vessels within the stroma. No necrosis or severe cellular atypia was observed (Fig. 5). The staining was performed by the Anatomic Pathology Laboratory of the General Hospital of Trikala. The thickness of the sections used was 5 µm, and the tissue sections were paraffin-embedded. The fixative used was buffered formalin 10%, at room temperature, for 36 h. The stain used was hematoxylin-eosin 0.5% alcohol (DIACEL), at room temperature and for a duration of 12 min. A LEICA DM2000 optical microscope was used. The thickness of the sections used in the immunohistochemical analysis was 4 µm, the sections were paraffin-embedded and dewaxed for 40 min at 75˚C. Immunohistochemistry was then performed with an automated BOND-LEICA system (Leica Biosystems), using the following protocol: i) Dewaxing with BOND^™^ Dewax solution,100% alcohol, BOND^™^ wash solution; ii) antigen retrieval: for CD10, BOND^™^ Epitope Retrieval ER2 Solution, HIER, was used for 20 min at 100˚C, while for smooth muscle actin (SMA), estrogen receptor (ER) and Ki67 antibodies used ER1 solution (pH 7) for 20 min; iii) the block peroxide kit (BOND) was used for 5 min; iv) only for CD10 antibody: Protein block solution was used for 20 min; v) primary antibody: dilution for CD10 (MENARINI) ready-to-use antibody (cat. no. 44 217 CD10 RTU), SMA (ZYTOMED) ready-to-use (cat. no. 1A4 A00002-IFU-IVD-0002), ER (DAKO) 1:40 (cat. no. M3643), Ki67 (Skytec) 1:150 for duration 30 min (MIB1 A00095-IFU-IVD, cat. no. CBB500), and post primary kit for duration 10 min at an incubation temperature of 100˚C; vi) secondary detection kit polymer for duration 10 min (no secondary antibodies were used herein); vii) DAB kit for duration 10 min, visualization; viii) counterstain: hematoxylin kit for duration 5 min at room temperature; ix) dehydration, mount section. The study of the slides was carried out using an optical microscope (LEICA DM2000; magnification, x40, x400 and x100). The following results were obtained: Ki67, 7%; SMA (+), weakly positive for ER, and negative for CD10 (Fig. 6). These findings excluded the diagnosis of fibrosarcoma.

Following a smooth post-operative course, the patient was discharged from the clinic on the fifth post-operative day. Upon the recommendation of urologists, the ureteral stents were removed 1 month after surgery. Upon a re-examination of the patient at the Gynecological Outpatient Clinic of the General Hospital of Trikala at 10 days and 3 months post-operatively, the patient was in excellent clinical condition and reported complete relief from the symptoms of abdominal distension with atypical abdominal pain, dyspepsia and anorexia. The results of blood tests were within normal ranges. A consultation for a re-examination at the Gynecological Outpatient Clinic of the General Hospital of Trikala at 6 months after surgery was made.

## Discussion

The diagnosis of intraligamental fibroids of uterus based on clinical manifestations is extremely difficult. In the majority of cases, intraligamental fibroids, even those that are large, are asymptomatic (4). Less commonly, they may manifest with more obvious symptoms, which are due to compression on the adjacent organs. Abdominal distension, frequent urination, constipation, anorexia, weight loss and pain in the ipsilateral to the tumor renal area are the main clinical manifestations that characterize huge intraligamental fibroids (5). In some cases, the pain may be acute and severe (9). Not unexpectedly, the patient described herein did not complain of any symptoms related to rectal or bladder compression from the huge intraligamental tumor. Abdominal distension, atypical abdominal pain and dyspepsia were the main symptoms in the patient in the present study. Possibly surprisingly, no hydronephrosis, due to right ureteric compression from the huge intraligamental fibroid, which is usually associated with large pelvic tumors, was observed (10).

Similarly, the pre-operative imaging diagnosis of intraligamental fibroids is difficult. Recently, there has been an attempt to evaluate magnetic resonance imaging findings in order to differentiate intaligamental fibroids from subserosal fibroids. Yajima *et al* (11) demonstrated that tumor shape, the attachment of the tumor to the uterus, ovary elevation on the side of the tumor and the separation of the round ligament from the ipsilateral uterine artery may be criteria for the differential diagnosis of intraligamental fibroids from subserosal fibroids. In the patient in the present study, neither computed tomography nor magnetic resonance imaging were able to establish the pre-operative diagnosis of intraligamental leiomyoma of the uterus. The effort made by the radiologists of the authors' hospital to differentiate subserosal fibroid from adnexal mass or intraligamental leiomyoma was not successful. The presence of a pedicle in the lateral wall of the uterus and the separation of the round ligament from the uterine artery proximal to the tumor were not imaged or not adequately assessed by the team of radiologists. In addition, the non-imaging of the right ovary was incorrectly not attributed to its elevation due to the displacement of the suspensory ligament by the presence of the huge intraligamental leiomyoma. Based on the experience from the management of patients with large intraligamental leiomyomas, it is considered that the determination and awareness of specific imaging features of these tumors may assist in the accurate preoperative diagnosis, which allows for the planning of the optimal surgical treatment and the avoidance of severe intraoperative and/or post-operative complications (12).

The diagnosis of intraligamental leiomyomas of the uterus in almost all cases, as in the patient described herein, is determined intraoperatively. Hysterectomy or myomectomy, depending on the age of the patient and the patient's desire to preserve fertility, is the main treatment option. The surgical treatment of intraligamental uterine leiomyomas is challenging. The understanding of the tumor characteristics (location, shape and size) and the proper pre-operative, intraoperative and post-operative management of these patients is critical for an optimal surgical outcome and for the prevention of potential intraoperative complications. Intraoperative hemorrhage is a common complication, which may be more prevalent during laparoscopic myomectomy, compared to myomectomy by laparotomy (4,5). Recently, however, in 2022, Wang *et al* (13) demonstrated that performing laparoscopic resection of a large intraligamental leiomyoma by combining two novel laparoscopic ligation techniques could be a safe and effective surgical treatment option, greatly reducing the risk of intraoperative hemorrhage and avoiding unintentional injury to adjacent organs, such as the bladder, rectum or ureters. In addition, the ureters can be protected from possible intraoperative damage by the pre-operative placement of ureteral stents (14).

Additionally, the scientific competence and skills of the surgical team that will be called to treat patients with large intraligamental fibroids are among the necessary requirements for the safe and successful outcome of myomectomy or hysterectomy with larapotomy or laparoscopy (5). In the patient in the present study, who did not wish to preserve fertility, abdominal total hysterectomy with bilateral adnexectomy was selected as the treatment of choice. Intraoperatively, the presence of a huge intraligamental leiomyoma was found, originating from the right lateral wall of the uterus with an extension in the broad ligament. The presence of multiple, various sized, subserosal and intramural uterine leiomyomas was also evident (Fig. 3, black arrows). It was decided to dissect and ligate the round ligament and the suspensory ligament, after the patient consented pre-operatively not to preserve the ovaries. In the case of myomectomy and in all cases of desire for ovarian preservation, the ligation of the suspensory ligament is not indicated. Subsequently, herein, the anterior leaf of the broad ligament was dissected with the aid of the electrothermal bipolar vessel sealing device, from the level of the upper third of the round ligament to the point of the attachment of the suspensory ligament with the ipsilateral ovary (Fig. 3, black line with double arrow), allowing access to the retroperitoneal space. The aid of the electrothermal bipolar vessel sealing device was critical in the dissection of the leiomyoma from the leaves of the broad ligament, to which it was attached with multiple, differently sized, feeding vessels. It is considered that the use of an electrothermal bipolar vessel sealing device greatly contributed to the control of intraoperative bleeding. The transfusion of two units of whole blood, which was deemed necessary for hemodynamic stabilization of the patient post-operatively, was the outcome of the low pre-operative hemoglobin. Careful dissection of the posterior leaf of the broad ligament from the leiomyoma and gentle surgical handling of the ureter was necessary in order to preserve adequate blood supply and avoid possible injury. Subsequently, following the ligation and dissection of the vascular pedicle of the leiomyoma from the right lateral wall of the uterus (Fig. 4, yellow arrows) and its resection from the surgical field, a total hysterectomy with bilateral adnexectomy was performed.

In conclusion, huge intraligamental fibroids of the uterus (>3,000 g) are extremely rare. A pre-operative diagnosis is difficult. Surgical treatment (myomectomy or hysterectomy) is challenging and requires well-organized medical centers and an experienced surgical team. The supplementary ligation of the suspensory ligament, in combination with the established ligation of the round ligament as a typical procedure for access to the retroperitoneal space, is estimated to further facilitate the resection of intraligamental fibroid and can greatly contribute to an optimal surgical outcome. However, it requires the resection of the ipsilateral ovary and for this reason, it should be avoided in patients who wish to preserve fertility and achieve future pregnancy. Finally, the present study highlights that the use of an electrothermal bipolar vessel sealing device appears to greatly contribute to reducing the risk of intraoperative blood loss.

## Data Availability

The datasets used and/or analyzed during the current study are available from the corresponding author on reasonable request.

## References

[1] Yang Q, Ciebiera M, Bariani MV, Ali M, Elkafas H, Boyer TG, Al-Hendy A (2022). Comprehensive review of uterine fibroids: Developmental origin, pathogenesis, and treatment. Endocr Rev.

[2] Zepiridis LI, Grimbizis GF, Tarlatzis BC (2016). Infertility and uterine fibroids. Best Pract Res Clin Obstet Gynaecol.

[3] Gomez E, Nguyen MT, Fursevich D, Macura K, Gupta A (2021). MRI-based pictorial review of the FIGO classification system for uterine fibroids. Abdom Radiol (NY).

[4] Huang PS, Sheu BC, Huang SC, Chang WC (2016). Intraligamental myomectomy strategy using laparoscopy. J Minim Invasive Gynecol.

[5] Lee CY, Chen CH (2021). Huge intraligamental leiomyoma: Two cases and review of the literature. Asian J Surg.

[6] Funaki K, Fukunishi H, Tsuji Y, Maeda T, Takahashi T (2013). Giant cystic leiomyoma of the uterus occupying the retroperitoneal space. J Radiol Case Rep.

[7] Rajanna DK, Pandey V, Janardhan S, Datti SN (2013). Broad ligament fibroid mimicking as ovarian tumor on ultrasonography and computed tomography scan. J Clin Imaging Sci.

[8] Naz Masood S, Masood Y, Mathrani J (2014). Diagnostic dilemma in broad ligament leiomyoma with cystic degeneration. Pak J Med Sci.

[9] Bechev B, Magunska N, Kovachev E, Ivanov S (2016). Laparoscopic treatment of intraligamental leiomyoma per magna. Akush Ginekol (Sofiia).

[10] Thanasa E, Thanasa A, Kamaretsos E, Paraoulakis I, Ziogas A, Kontogeorgis G, Grapsidi V, Gerokostas EE, Kontochristos V, Thanasas I (2023). Large cervical leiomyoma of the uterus: A rare cause of chronic pelvic pain associated with obstructive uropathy and renal dysfunction: A case report. Cureus.

[11] Yajima R, Kido A, Kuwahara R, Moribata Y, Chigusa Y, Himoto Y, Kurata Y, Matsumoto Y, Otani S, Nishio N (2021). Diagnostic performance of preoperative MR imaging findings for differentiation of uterine leiomyoma with intraligamentous growth from subserosal leiomyoma. Abdom Radiol (NY).

[12] Ambrosio M, Raimondo D, Savelli L, Salucci P, Arena A, Borghese G, Mattioli G, Giaquinto I, Scifo MC, Meriggiola MC (2020). Transvaginal Ultrasound And Doppler Features Of Intraligamental Myomas. J Ultrasound Med.

[13] Wang S, Wang D, Zhao F (2022). A combination of two novel ligation techniques for complicated laparoscopic Intraligamental myomectomy. Fertil Steril.

[14] Yanagisawa T, Mori K, Quhal F, Kawada T, Mostafaei H, Laukhtina E, Rajwa P, Sari Motlagh R, Aydh A, König F (2023). Iatrogenic ureteric injury during abdominal or pelvic surgery: A meta-analysis. BJU Int.

